# Synthesis and biological function of Nickel and Copper nanoparticles

**DOI:** 10.1016/j.heliyon.2019.e01878

**Published:** 2019-06-06

**Authors:** Jyoti Chaudhary, Giriraj Tailor, B.L. Yadav, Oshon Michael

**Affiliations:** a Department of Polymer Science, M.L.S. University, Udaipur, Rajasthan, India; b Department of Botany Mewar University, Gangrar, Chittorgarh, Rajasthan, India; c Department of Chemistry, Mewar University, Chittorgarh, Rajasthan, India

**Keywords:** Nanotechnology, Materials chemistry, Antimicrobial nanoparticles, Monoclinic, XRD, ZOI

## Abstract

Nickel and Copper nanoparticles were synthesized by simple chemical method and studied for antimicrobial activities. The size of synthesized Nickel and Copper nanoparticles was found to be 24.00 nm and 13.13 nm respectively. The XRD analysis reveals the crystal system of Nickel and Copper nanoparticles. Copper nanoparticles were found orthorhombic whereas the nickel nanoparticles were monoclinic. The antimicrobial activities of Nickel and Copper nanoparticles dispersed in DMSO was investigated. Comparative sensitivity test of these synthesized nanoparticles was carried out against three pathogenic micro-organisms (Gram negative bacteria), viz. *Escherichia coli, Klebsiella pneumoniae* and *Pneumonia Typhus,* using agar diffusion cup plate method. Copper and Nickel nanoparticles have shown appreciable sensitivity at 100 μg/ml against all test micro-organisms. Comparatively, Copper nanoparticles were found to exhibit higher zone of inhibition (ZOI) than Nickel nanoparticles.

## 1 Introduction

Metallic nanoparticles (NPs) are known for potential application in catalysis, electronics, optoelectronics, information stock, biosensors, and surface enhanced Raman spectroscopy (SERS), their synthesis and properties have been studied in depth by several workers [1, 2, 3, 4, 5, 6, 7, 8, 9, 10]. These particles have a wide functional diversity and can exist as common structural elements or composites compared to bulk materials. The physico-chemical properties of metallic nanoparticles are mostly rated by the parameters like size, shape, and composition [11, 12, 28]. Metallic nanoparticles of exclusive sizes and morphologies can be readily synthesized using chemical and physical methods [29, 30]. Most of the methods use toxic chemicals as reducing agents, organic solvents and non-biodegradable stabilizing agents which are potentially dangerous to the environment and biological systems. Moreover, most of these methods are complicated and involve non-standard conditions making them quite expensive. Thus the biosynthesis of nanoparticles is being proved as a cost effective environmental friendly alternative to chemical and physical methods [31, 32, 33, 34, 35, 36]. Consequently, micro-organisms and plant extracts have been used in synthesis of nanomaterials [24, 25]. The use of plant extracts for synthesis of nanoparticles is profitable over microorganisms due to elaborate process of maintaining cell cultures. The synthesis of metal nanoparticles using plant extracts has provided a rapid, cost-effective biosynthetic protocol for bulk synthesis of stable metallic nanoparticles. In the presentwork an attempt has been made to develop green synthesis of metal nanoparticles through a single-step, room-temperature from metal ions using environmentally benign reagents [13, 14].

The noble metals such as gold and silver have been used in synthesis of nanoparticles. Now a day, copper (Cu) and nickel (Ni) are being used instead of noble metals in synthesis of metallic nanoparticles as they are more economical than gold and silver. Recent investigations have been extended to the study of other metals, such as Cu and Ni, that could have antibacterial activity. However, little attention has been paid to the study of bimetallic Cu–Ni NPs, although some studies have already shown some features of alloy NPs that distinguish them from the pure ones [15]. Cu NPs [16, 17, 18, 19] and copper oxide NPs [20, 21] have reported to have antimicrobial activity. Similar findings have been reported for Ni NPs. Many studies have shown that Cu and Ni NPs have bactericidal activities [26, 27]. Nevertheless, they have not been synthesized in aqueous solution without using stabilizers as polymers, ligands, salts, etc. That can hinder their properties. Finally, it is important to note that until now, the anti microbial properties of Cu–Ni bimetallic NPs have not been studied. Therefore, the purposes of this study were the synthesis and characterization of Cu, Ni to investigate their antimicrobial activity.

## 2 Materials and methods

During the present study AR grade chemicals such as Phenol, formaldehyde and acetic acid (Central Drug House Pvt. Limited) and hydrochloric acid (Fisher Scientific) were used. Metal ion solutions have been prepared by dissolving suitable amount of metal salt in distilled water. Copper and Nickel nanoparticles have been prepared by simple chemical precipitation method and characterisation was done by SEM, thermal gravimetric analysis and infrared spectroscopy. The synthesis of metallic nanoparticles is a twostep process. The first step includes the synthesis of polymer metal complex and second step the synthesis of metallic nanoparticles. The detailed method of synthesis of metallic nanoparticles has already been given in our earlier publication [22]. However, the process of synthesis of Nickel and Copper nanoparticles has been presented below in the form of flow chart:

### 2.1 Synthesis of nickel nanoparticles

**Figure:**
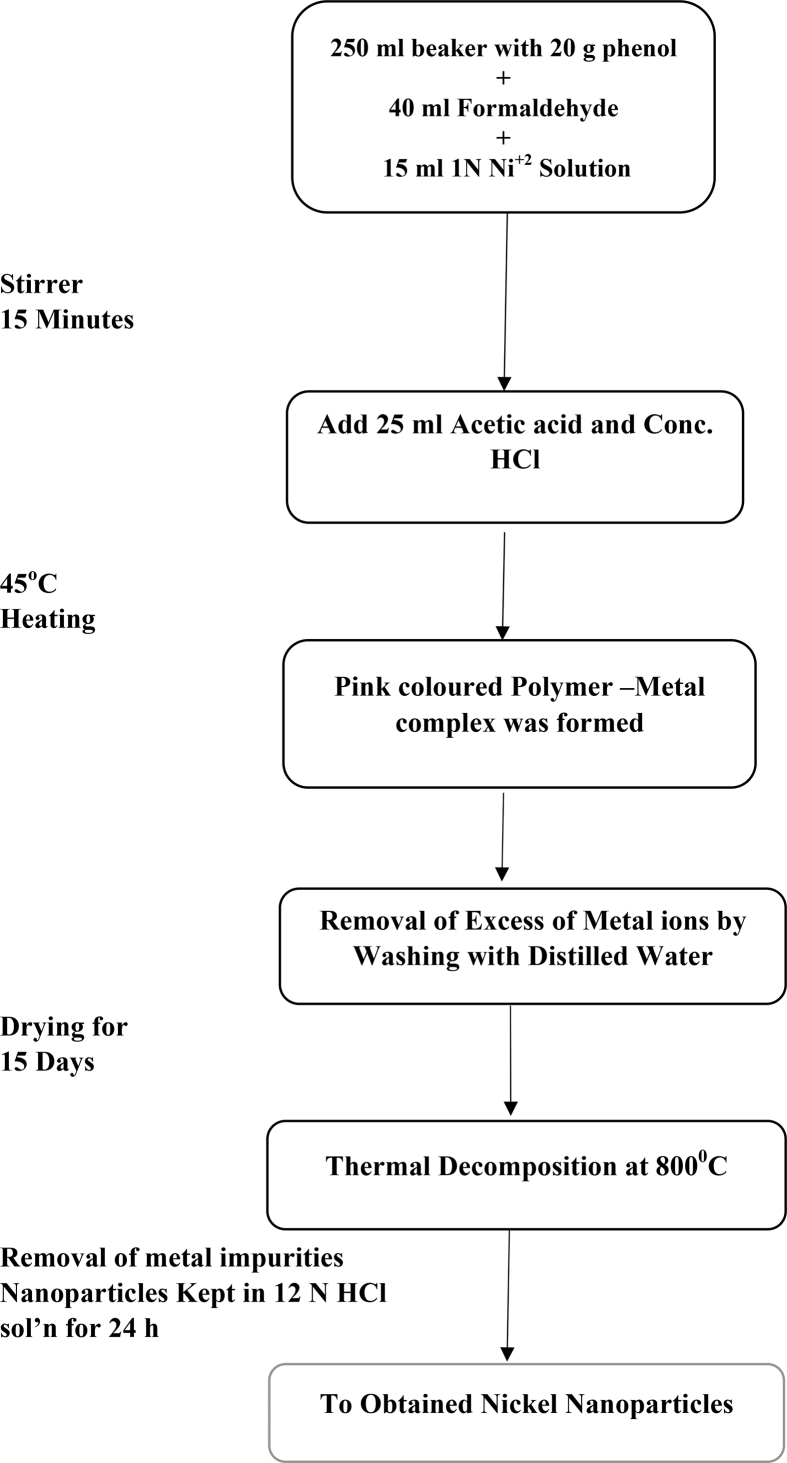

### 2.2 Synthesis of copper nanoparticles

**Figure:**
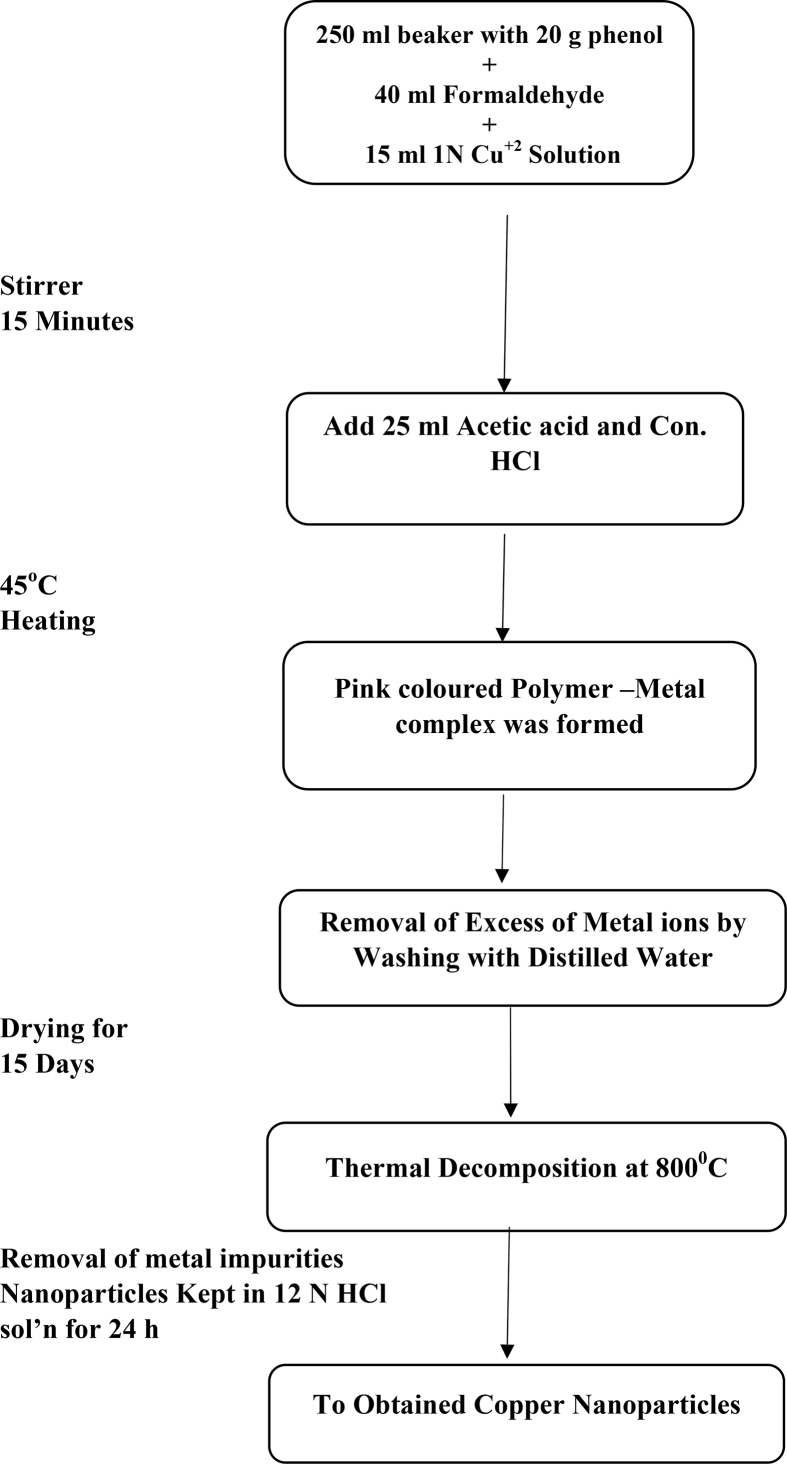

### 2.3 Antimicrobial activity

Comparative sensitivity test of Copper and Nickel nanoparticles was carried out against three pathogenic micro-organisms, namely, *E. coli, K. pneumoniae and P. typhus,* using agar diffusion cup plate method. Luria agar media was prepared and used for this purpose. Both nanoparticles were screened for antimicrobial activity at 100 μg/ml concentration. Ciprofloxacin was used as control. 0.001g each of Copper and Nickel nanoparticles were accurately weighed and dissolved in 10ml of DMSO. 100 μl of each stock solution was diluted with 900 μl of DMSO, to get a concentration of 100 μg/ml. Luria agar medium was prepared appropriately and dispensed (half filled) into six sterile petri dishes, and allowed for about 1 hour to gel uniformly. After gelling, the petri-dishes were inoculated with 50μl of each test micro-organism and uniformly spread with a spreader and labelled accordingly. Well diffusion method was followed for sensitivity test against the pathogenic bacteria. Wells of 7 mm in diameter were made with the help of a sterile cork borer in each petri dish. 100 μg/ml concentration of each of copper/nickel nanoparticle solution was dispensed (half filled) into the wells. The petri plates were incubated at 370C for 24 hours. The zone of inhibition was measured in millimetre (mm).

## 3 Results and discussion

### 3.1 X-RD characterization

X – RD diffraction pattern provides information's on size and shape of the unit cell from peak positions and information on electron density within the unit cell. Orthorhombic copper nanoparticles have been determined using XRD technique. Applying Debye – Scherrer equation to the XRD pattern of the copper nanoparticles, it has been found that the average size of nanoparticles is 13.13 nm, bravais lattice is primitive, space group is pccn (56) and 2ϴ = 42.045 [22].

XRD pattern showed that the size of nickel nanoparticles was 24.0 nm and has maximum intensity diffraction peak at 2ϴ = 12.50290 which indicate the presence of crystalline structure and the crystal system is monoclinic [23].

### 3.2 Antimicrobial activity

**Fig. 1:**
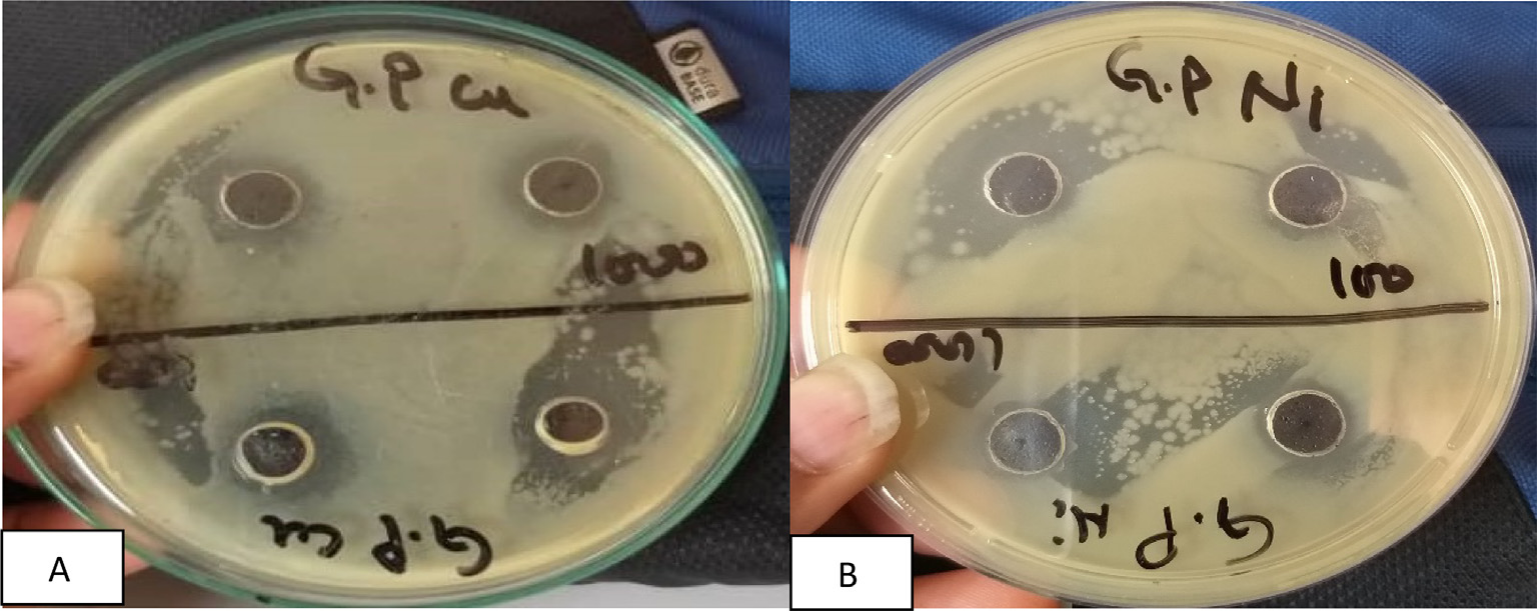
A-B Zone of inhibition of Copper and Nickel nanoparticles respectively against *P. typhus*.

**Fig. 2:**
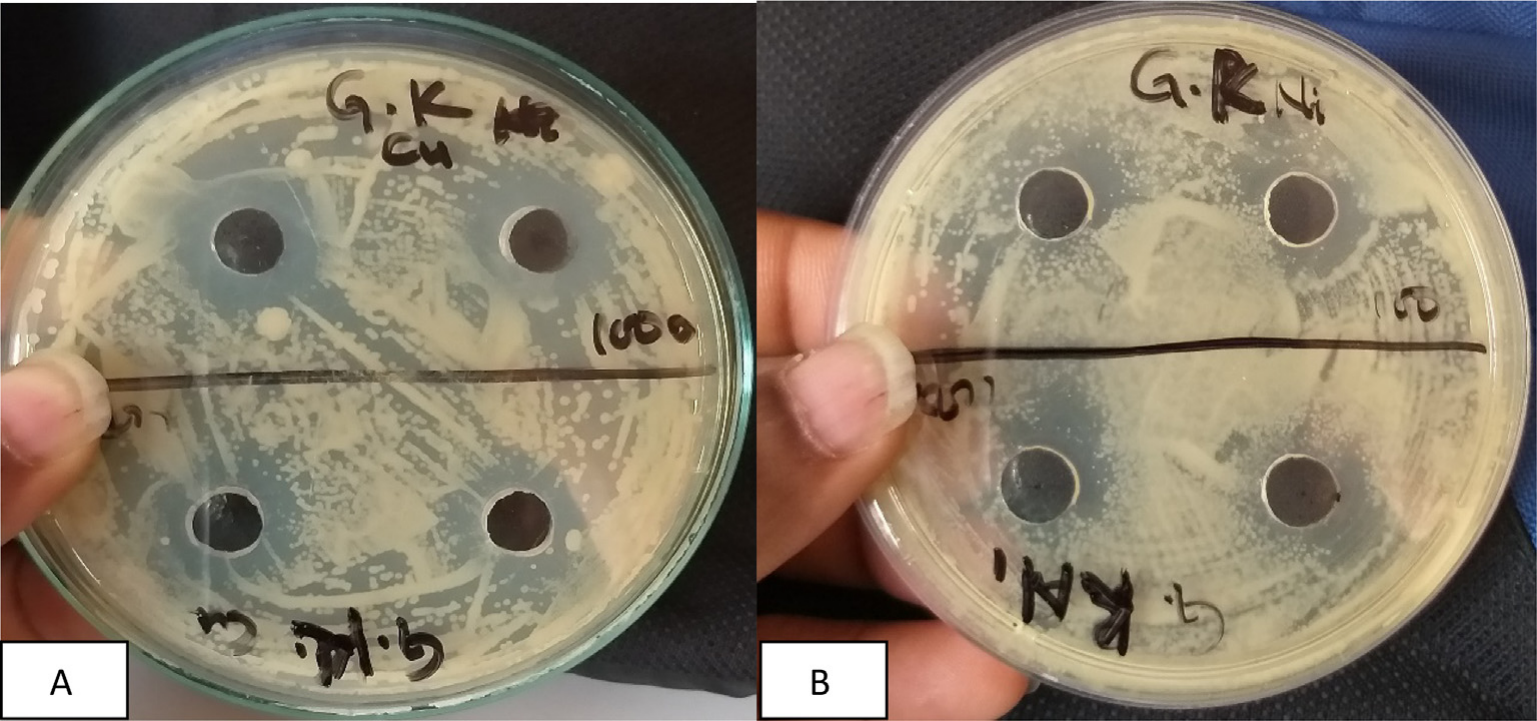
A-B Zone of inhibition of Copper and Nickel nanoparticles respectively against *K. pneumoniae*.

**Fig. 3:**
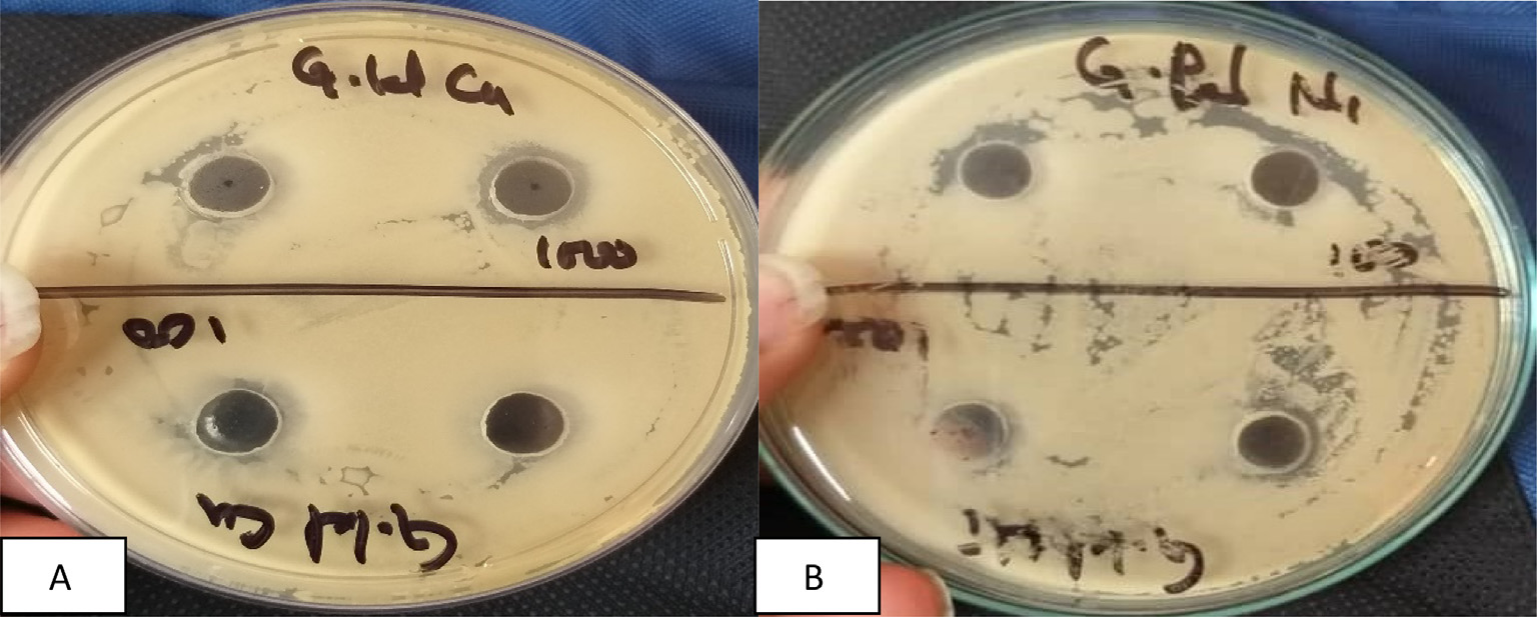
A-B Zone of inhibition of Copper and nickel Nanoparticles respectively against *E. coli*.

Observations on the zone of inhibition of Nickel and Copper nanoparticles against the three pathogenic bacteria have been presented in Table 1 and Figs. 1, 2, and 3.

**Table 1:** Zone of Inhibition (mm) at 100 μg/ml of Copper and Nickel nanoparticles.

Nanoparticles	*E. coli*	*K. pneumoniae*	*P. typhus*
Copper	5.0	9.0	6.0
Nickel	4.0	7.0	4.0

The data presented in table – 1 reveal that both copper and nickel nanoparticles exhibit highest ZOI against *Klebsiella pneumonie* (Fig. 2) However, the ZOI of nickel nanoparticles is comparatively lower (7.0 mm) than copper nano-composite (9.0 mm). Zone of inhibition of copper nanoparticles against *E. coli and Pneumonia Typhus* are comparatively higher than those shown by nickel nanoparticles (Figs. 1 and 3). Nickel nanoparticles are more effective against *K*. *Pneuminiae* than the other two bacteria. Thus, copper nanoparticles are more effective against *K*. *pneuminiae*, *P. typhus* and *E. coli* than nickel nanoparticles. The sensitivity of the microorganisms to the copper nanoparticles may be arranged starting with *K. Pneumonia* (9.0 mm) followed by *P. typhus* (6.0 mm) and *E. Coli* (5.0 mm) in descending order. The higher ZOI in case of *E. coli* by the copper nanoparticles synthesized by electrolysis method was reported earlier [24] and an efficient antimicrobial activity of nickel nanoparticles against pathogenic bacteria has also been reported earlier (Chaudhary et al 2015). Values of Control.

Heavy metals are toxic and reactive with of proteins and produce the significant increase in permeability affecting transport system through plasma membrane as a result of which bacterial cell becomes unable to regulate the transport across the plasma membrane resulting into the death of bacteria [23].

The present paper reports a simple, fast, effective polymer based technique to synthesize copper and nickel nanoparticles using thermosetting polymer and copper and nickel metal salt as a precursor. Nanoparticles synthesized by the simple chemical precipitation method. XRD analysis proves the nanostructure of the copper and nickel nanoparticles through its crystal analysis and spacing pattern. The antibacterial activity of copper and nickel nanoparticles was compared for various micro-organisms using the agar diffusion cup plate method. The diameter of inhibition zone (DIZ) reflects magnitude of susceptibility of the micro-organism. Both copper and nickel nanoparticles showed appreciable sensitivity at 100 μg/ml against all test micro-organisms. Comparatively, copper nanoparticles showed higher zone of inhibition (ZOI) than nickel nanoparticles.

## Declarations

### Author contribution statement

Jyoti Chaudhary: Contributed reagents, materials, analysis tools or data.

Giriraj Tailor: Conceived and designed the experiments; Analyzed and interpreted the data; Wrote the paper.

Oshon Michal: Performed the experiments.

B.L. Yadav: Analyzed and interpreted the data.

### Funding statement

This research did not receive any specific grant from funding agencies in the public, commercial, or not-for-profit sectors.

### Competing interest statement

The authors declare no conflict of interest.

### Additional information

No additional information is available for this paper.

## References

[1] Li C.-H., Li M.-C., Liu S.-P., Jamison A.C., Lee D., Lee T.R., Lee T.-C. (2016). Plasmonically enhanced photocatalytic hydrogen production from water: the critical role of tunable surface plasmon resonance from gold-silver nanoshells. ACS Appl.Mater. Inter..

[2] Hien Pham T.T., Cao C., Sim S.J. (2008). Application of citrate-stabilized gold-coated FerricOxide composite nanoparticles for biological separations. J. Magn. Magn. Mater..

[3] Li J.J., Peng X. (2004). Photocatalytic activity of gold nanocrystals and its role inDeterminingthe stability of surface thiol monolayers. J. Nanosci. Nanotechnol..

[4] Tessier P.M., Velev O.D., Kalambur A.T., Rabolt J.F., Lenhoff A.M., Kaler E.W. (2000). Assembly of gold nanostructured films templated by colloidal crystals and use insurface-enhanced Raman spectroscopy. J. Am. Chem. Soc..

[5] Kim S.-W., Kim M., Lee W.Y., Hyeon T. (2002). Fabrication of hollow palladium spheres andTheir successful application as the recyclable heterogeneous catalyst for SuzukiCoupling reactions. J. Am. Chem. Soc..

[6] Nicewarner-Pena S.R., Griffith Freeman R., Reiss B.D., He L., Pena D.J., Walton I.D., Cromer R., Keating C.D., Natan M.J. (2001). Submicrometer metallic barcodes. Science.

[7] Chen J., Saeki F., Wiley B.J., Cang H., Cobb M.J., Li Z.-Y., Au L., Zhang H., Kimmey M.B., Li Xia. (2005). Gold nanocages: bioconjugation and their potential use asOptical imaging contrast agents. Nano Lett..

[8] Song L., Mao K., Zhou X., Hu J. (2016). A novel biosensor based on Au@Ag core–shell nanoparticles for SERS detection of arsenic (III). Talanta.

[9] Varghese R., Almalki M.A., Ilavenil S., Rebecca J., Choi K.C. (2017). Silver nanopaticles synthesized using the seed extract of trigonellafoenum-Graecum L. And TheirAntimicrobial mechanism and anticancer properties. Saudi J. Biol. Sci..

[10] Li X., Liu H., Liu S., Zhang J., Chen W., Huang C., Mao L. (2016). Effect of Pt–Pd hybridnano-particle on CdS's activity for water splitting under visible light. Int. J. Hydrogen Energy.

[11] Logunov S.L., Ahmadi T.S., El-Sayed M.A., Khoury J.T., Whetten R.L. (1997). Electron dynamics of passivated gold nanocrystals probed by subpicosecond transient absorption spectroscopy. J. Phys. Chem. B.

[12] Burda C., Chen X., Narayanan R., El-Sayed M.A. (2005). Chemistry and properties of nanocrystals of different shapes. Chem. Rev..

[13] Bogunia-Kubik K., Sugisaka M. (2002). From molecular biology to nanotechnology and nanomedicine. Biosystems.

[14] Perez J., Bax L., Escolano C. (2005). Roadmap Report on Nanoparticles.

[15] Ferrando R., Jellinek J., Johnston R.L. (2008). Nanoalloys:  from theory to applications of alloy clusters and nanoparticles. Chem. Rev..

[16] Esteban-Cubillo A., Pecharromán C., Aguilar E., Santarén J., Moya J.S. (2006). J. Antibacterial activity of copper monodispersed nanoprticles into sepioliteMater. Science.

[17] Raffi M., Mehrwan S., Bhatti T.M., Akhter J.I., Hameed A., Yawar W., ulHasan M.M. (2010). Investigations into the antibacterial behaviour copper nanoparticles. Ann. Microbiol..

[18] Cioffi N., Torsi L., Ditaranto N., Tantillo G., Ghibelli L., Sabbatini L., Bleve-Zacheo T., DAlessio M., Zambonin P.G., Traversa E. (2005). Copper nanoparticle/polymer composites with antifungal and bacteriostatic properties. Chem. Mater..

[19] Chatterjee A.K., Sarkar R.K., Chattopadhyay A.P., Aich P., Chakraborty R., Basu T. (2012). A simple robust method for synthesis of metallic copper nanoparticles of high antibacterial potency against E. coli. Nanotechnol..

[20] Gabbay J., Borkow G., Mishal J., Magen E., Zatcoff R., Shemer-Avni Y. (2006). Copper oxide impregnated textiles with potent biocidal activities. J. Ind. Text..

[21] Ren G., Hu D., Cheng E.W.C., Vargas-Reus M.A., Reip P., Allaker R.P. (2009). Synthesis, characterization and antibacterial activity of copper, nickel and bimetallic Cu–Ni nanoparticles for potential use in dental materials. Int. J.Antimicrob.Agents.

[22] Chaudhary J., Tailor G., Kumar D., Joshi A. (2017). Synthesis and thermal properties of copper nanoparticles. Asian J. Chem..

[23] Chaudhary J., Tailor G., Kumar D., Shailesh S.K. (2016). Synthesis and structural study of nickel (II) bakelite nanocomposite by X-ray diffaraction. Int. J. Metall. Mater. Sci. Eng..

[24] Subhankari I., Nayak P.L. (2013). Antimicrobial activity of copper nanoparticles synthesised by Ginger (zingiber officinale) extract. World J. Nucl. Sci. Technol..

[25] Pandian C.J., Palanivel R., Dhanasekaran S. (2016). Screening antimicrobial activity of nickel nanoparticles synthesized using *Ocimum sanctum* leaf extract. J. Nano..

[26] Lee H.J., Song J.Y., Kim B.S. (2013). Biological synthesis of copper nanoparticles using *Magnolia kobus* leaf extract and their antibacterial activity. J. Chem. Technol. Biotechnol..

[27] Figueroa L.A., Luckie R.M., Vilchis R.S., Mejía O.F.O. (2014). Synthesis, characterization and antibacterial activity of copper, nickel and bimetallic Cu–Ni nanoparticles for potential use in dental materials. Prog. Nat. Sci.Mater. Int..

[28] Esmaeili E., Salavati Niasaria M., Mohandes F., Davara F., Seyghalkarb H. (2011). Modified single-phase hematite nanoparticles via a facile approach for large-scale synthesis. Chem. Eng. J..

[29] Salavati-Niasari M., Davar F., Mazaheri M. (2009). Synthesis and characterization of Zns Nanocluster via hydrothermal processing from [Bis (salicylidene) zinc (II)]. J. Alloy. Comp..

[30] Salavati-Niasari M., Salemi P., Davar F. (2005). Oxidation of cyclohexene with *tert*- butylhydroperoxide and hydrogen peroxide catalysted by Cu(II), Ni(II), Co(II) and Mn(II) complexes of *N,N*′-bis-(*α*-methylsalicylidene)-2,2-dimethylpropane-1,3-diamine, supported on alumina. J. Mol. Catal. A Chem..

[31] Kianpour G., Salavati-Niasari M., Emadi H. (2013). Sonochemical synthesis and characterization of NiMoO4 nanorods. Ultrason. Sonochem..

[32] Salavati-Niasari M. (2005). Nanoscale microreactor-encapsulation of 18-membered decaaza macrocycle nickel(II) complexes. Inorg. Chem. Commun..

[33] Salavati-Niasari M. (2005). Nanodimensional microreactor-encapsulation of 18-membered decaaza macrocycle copper(II) complexes. Chem. Lett..

[34] Salavati-Niasari M., Dadkhah M., Davar F. (2009). Synthesis and characterization of purecubic zirconium oxide nanocrystals by decomposition of bis-aqua, tris- acetylacetonato zirconium(IV) nitrate as new precursor complex. Inorg. Chim. Acta.

[35] Salavati-Niasari M., Farzaneh F., Ghandi M. (2002). Oxidation of cyclohexene with tert- butylhydroperoxide and hydrogen peroxide catalyzed by alumina-supported manganese(II) complexes. J. Mol. Catal. A Chem..

[36] Zinatloo-Ajabshir S., Salavati-Niasari M., Hamadanian M. (2015). Praseodymium oxide nanostructures: Novel Solvent-Less Preparation, Characterization and Investigation of their optical and Photocatalytic Properties.

